# Hymenoptera of Galápagos: diversity and distribution of one of the most species-rich insect orders in the archipelago

**DOI:** 10.3897/zookeys.1294.200001

**Published:** 2026-10-09

**Authors:** Patricio Picón-Rentería, Henri W. Herrera, José M. Avendaño, María C. Tocora-Protz, Andrés F. Sánchez Restrepo, Fernando Fernández, John M. Heraty, Ashley Y. Bui, Stewart Peck, Carlos E. Sarmiento

**Affiliations:** 1 Museo de Entomología, Facultad de Recursos Naturales, Escuela Superior Politécnica de Chimborazo, Riobamba, 060101, Ecuador Museo de Entomología, Facultad de Recursos Naturales, Escuela Superior Politécnica de Chimborazo Riobamba Ecuador; 2 Laboratorio de Sistemática y Biología Comparada de Insectos, Instituto de Ciencias Naturales, Facultad de Ciencias, Universidad Nacional de Colombia, Sede Bogotá, Bogotá, 111321, Colombia Laboratorio de Sistemática y Biología Comparada de Insectos, Instituto de Ciencias Naturales, Facultad de Ciencias, Universidad Nacional de Colombia Bogotá Colombia; 3 Charles Darwin Research Station, Charles Darwin Foundation, Puerto Ayora, Santa Cruz, Galápagos, 200350, Ecuador Charles Darwin Research Station, Charles Darwin Foundation Galápagos Ecuador; 4 Department of Ecology and Evolutionary Biology, University of Toronto, Toronto, ON M5S 3B2, Canada Department of Ecology and Evolutionary Biology, University of Toronto Toronto Canada; 5 Department of Biological Sciences, Texas Tech University, Lubbock, TX 79409, USA Department of Biological Sciences, Texas Tech University Lubbock United States of America; 6 Fundación para el Estudio de Especies Invasivas (FuEDEI), Hurlingham, Buenos Aires, B1686, Argentina Fundación para el Estudio de Especies Invasivas (FuEDEI) Hurlingham Argentina; 7 Consejo Nacional de Investigaciones Científicas y Técnicas (CONICET), Buenos Aires, Argentina Consejo Nacional de Investigaciones Científicas y Técnicas (CONICET) Buenos Aires Argentina; 8 Instituto de Ciencias Naturales, Facultad de Ciencias, Universidad Nacional de Colombia, Sede Bogotá, Bogotá, 111321, Colombia Instituto de Ciencias Naturales, Facultad de Ciencias, Universidad Nacional de Colombia Bogotá Colombia; 9 Department of Entomology, University of California, Riverside, Riverside, CA 92521, USA Department of Entomology, University of California, Riverside Riverside United States of America; 10 Department of Biology, Carleton University, Ottawa, ON K1S 5B6, Canada Department of Biology, Carleton University Ottawa Canada

**Keywords:** Archipelago ecology, biodiversity databases, community composition, Ecuador, island biogeography, species diversity

## Abstract

Despite the importance of the Galápagos archipelago as a key site for the study of biodiversity and evolution, significant components of its invertebrate biodiversity, particularly Hymenoptera, remain insufficiently documented. Occurrence records of Hymenoptera from 1959–2025 were combined from structured surveys of non-formicid Hymenoptera, long-term ant sampling, museum collections, literature, and online sources to assess the richness, distribution, and species composition of hymenopterans across the archipelago. Based on 21,364 records, 524 species/morphospecies, including 137 species, one subspecies, and 386 morphospecies are documented. These records include 20 newly recorded species and 134 newly recorded genera for Galápagos, representing 11 previously unrecorded families. These records substantially increase previous estimates, with the greatest additions observed in Chalcidoidea and Ichneumonoidea. Species accumulation curves and incidence-based richness estimators (Bootstrap = 595.47, Jackknife 1 = 673.38, Chao2 = 658.26) indicate that additional species/morphospecies may remain undocumented. Species/morphospecies richness was positively associated with island area after accounting for sampling effort. Species composition showed partial similarity among some islands and overlap among the littoral, dry, and transition zones; the humid zone contained the highest proportion of unique species/morphospecies. A considerable proportion of the named species were classified as introduced. Overall, this study establishes an updated baseline for future Hymenoptera research in Galápagos and provides a framework for evaluating biogeographic patterns in oceanic island systems.

## Introduction

Despite historic efforts to document global biodiversity, many ecosystems and taxonomic groups remain extensively unexplored (Colwell and Coddington 1994; Antonelli et al. 2018). These gaps create significant challenges in recognizing the richness of specific groups (Jarnevich et al. 2006), understanding ecological and macroevolutionary patterns (Diniz-Filho et al. 2010), and developing well-supported conservation initiatives (Mooney et al. 2009; Thomson et al. 2018; Theng et al. 2020). Detailed taxonomic inventories are crucial for identifying areas where further sampling efforts should be prioritized (Herrera et al. 2024) and for tracking invasive species (Picón-Rentería et al. 2024) and also provide a better understanding of the spatial distribution and biogeography of species (Maestri and Patterson 2016; Toussaint et al. 2017), as well as patterns of endemism (Gentry 1992; Crother and Murray 2011; Aagesen et al. 2013).

The Galápagos archipelago looms large in the history of biological thinking, harbors unique biodiversity, and serves as a living laboratory (Harpp et al. 2014). Like many oceanic islands, this archipelago is a target for species protection due to its high levels of endemism and susceptibility to human disturbance (Russell and Kueffer 2019). Several endemic species are known from the Galápagos, including giant tortoises, marine and pink land iguanas, and Darwin’s finches (Parent et al. 2008; Gentile and Snell 2009; Grant 2017; Gaughran et al. 2018). Despite these precedents, diversity remains largely unexplored in some taxa. For instance, although current records of insects represent 51% of the biodiversity in the Galápagos (Charles Darwin Foundation 2025a), detailed knowledge of this fauna and also of deep-sea benthic environments has only recently increased (Salinas-de-León et al. 2020; Sinclair 2023; Herrera et al. 2024).

For Insecta, we have reliable lists for Coleoptera comprising 486 species (Peck 2006), Diptera with 324 species (Sinclair 2023), and Lepidoptera with 311 species (Roque-Albelo and Landry 2016). Although Hymenoptera are considered one of the most species-rich insect orders globally (Polaszek and Vilhemsen 2023), this global pattern does not translate into reliable local estimates for the Galápagos, where the reported numbers of species are not comparable to other orders. In 1966, Linsley and Usinger reported only 31 Hymenoptera species, of which 28 had a binomen (Linsley and Usinger 1966). The reported diversity has been mostly represented by ants (50 species; Herrera et al. 2020, 2024), whereas parasitoid wasps remained comparatively underrepresented in available inventories (Causton et al. 2006). Peck et al. (1998) later mentioned 253 species of Hymenoptera but did not provide an associated taxonomic list. Posteriorly, a preliminary list of 84 named species was provided by Heraty and Herrera (2017). Recent efforts have improved our knowledge of hymenopteran diversity in the Galápagos (Fernández et al. 2018; Camacho-Erazo et al. 2024; Sandoval-B et al. 2024), increasing the total number of named species to 111; however, this number is expected to be much higher given the worldwide diversity of the group; the richness of Lepidoptera, Coleoptera, and Diptera, which include many potential hosts of parasitoid Hymenoptera; and the historical lack of knowledge of microhymenopteran diversity in the islands.

Here, we provide a comprehensive list of the Hymenoptera in the Galápagos and assess patterns of richness and distribution across the archipelago. We compiled an integrated dataset integrating structured field surveys, non-structured sampling, long-term ant sampling, museum collections, literature, and online sources. In addition to documenting diversity, we evaluated spatial patterns of species richness across islands and vegetation zones. We also characterized taxon accumulation across the sampled geographic extent and analyzed patterns of taxonomic and geographic coverage derived from multiple data sources. This study provides an updated taxonomic and distributional baseline for Hymenoptera in the Galápagos.

## Materials and methods

### Study design, sampling, and data collection

**Figure 1.**
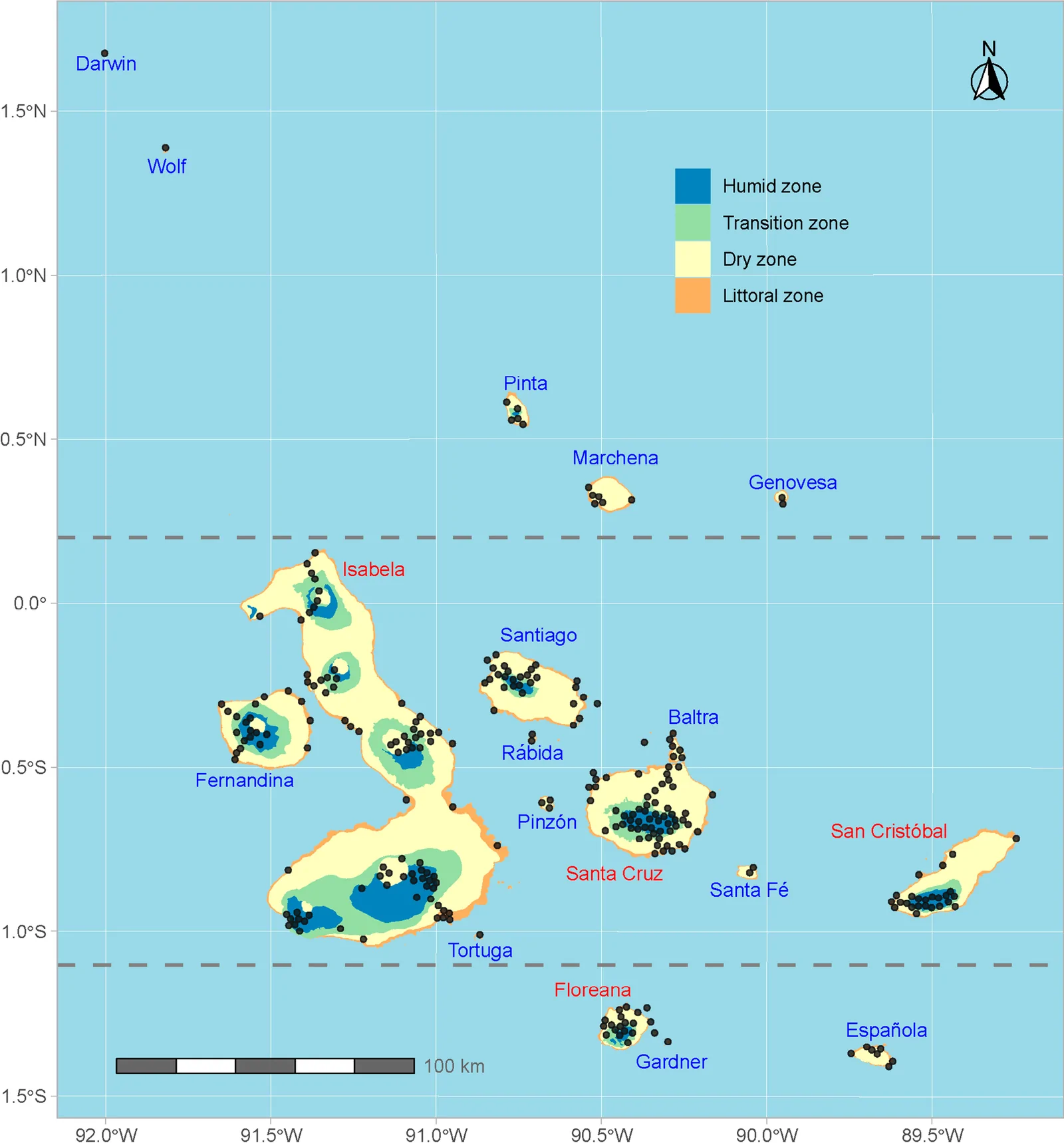
Distribution of mapped localities represented by records with geographic coordinates in the four datasets: wasp dataset, ant dataset, museum dataset, and literature dataset. Island names shown in red indicate inhabited islands. Vegetation zone classification follows Jackson (2006).

We generated an integrated dataset of Hymenoptera records for the Galápagos Islands by compiling data from four groups of sources: (1) wasp dataset: 2,424 records, including 2,414 records from an extensive structured sampling survey of non-formicid Hymenoptera conducted by the authors in 2018, 2019, and 2021, and ten additional opportunistic records from 2025; (2) ant dataset: a long-term survey of ants (*n* = 15,502); (3) museum dataset (*n* = 3,305), comprising records from the University of California, Riverside Entomology Research Museum (UCRC_ENT, *n* = 2,065), primarily Chalcidoidea from expeditions by Stewart Peck and others from 1985–1996, the Charles Darwin Research Station Collection (ICCDRS, *n* = 1,167), the California Academy of Sciences (CASC, *n* = 34), the Canadian National Collection of Insects, Arachnids, and Nematodes (CNC, *n* = 24), the Australian National Insect Collection (ANIC, *n* = 6), the Natural History Museum (NHMUK, formerly BMNH, *n* = 4), the United States National Museum (USNM, *n* = 3), the Bavarian Natural History Museum (ZSMC, *n* = 1), and one record without an institution code; and (4) literature dataset: 133 records compiled from Galápagos arthropod fauna surveys, systematic revisions, online sources, and papers referring to Hymenoptera reported from the islands. The taxonomic information associated with these records was compiled and standardized in a checklist (Suppl. material 1). The four source datasets were integrated into a single integrated dataset (Suppl. material 2). All georeferenced records were combined to represent the overall spatial coverage of Hymenoptera records across the archipelago (Fig. 1). The four source datasets differed in their spatial coverage among islands (Suppl. material 3: fig. S1A–D). Here, a record refers to an occurrence represented by a single row in the integrated dataset, with information such as taxonomic identification, locality, date, or sampling event.

The structured sampling component of the wasp dataset was conducted on the ten largest islands of the Galápagos archipelago (Suppl. material 3: fig. S1B): Santa Cruz, Fernandina, and Isabela (Darwin and Alcedo volcanoes) in 2018; San Cristóbal, Isabela (Sierra Negra volcano), and Floreana in 2019; and Genovesa, Marchena, Pinta, Santiago, and Española in 2021. Sampling was conducted in the littoral, dry, transition, and humid vegetation zones (Fig. 1; Jackson 2006). In each zone, a sampling site comprised three Malaise traps separated by 50 m and operated for eight days, five yellow traps placed 5 m from each Malaise trap and operated for 72 h, four transects of 50 double net sweeps, and an unspecified number of ad libitum collection events using nets, forceps, or aspirators. The total sampling effort was 146 Malaise units, 208 yellow traps, and 174 net sweeps. Additional opportunistic collections conducted in 2025 on Pinzón and Rábida islands and Edén Islet consisted of 24-h Malaise trapping and ad libitum sweep-net collecting. Records from the 2025 sampling were incorporated only into the taxonomic inventory and excluded from summaries and comparisons of the structured sampling survey. For Formicidae, the ant dataset included long-term sampling using several methods, including pitfall traps, baits, aspirators, manual collection, pan traps, Winkler extraction, Malaise traps, sticky traps, and sweep nets, across 51 islands, islets, and rocks from 2001 to 2012 (Herrera et al. 2024). All specimens represented in the wasp dataset are deposited in the Museum of Entomology of the Escuela Superior Politécnica de Chimborazo (ECESPOCH).

### Specimen identification

Specimens of Hymenoptera other than Formicidae were identified to the family level following available taxonomic keys (Goulet and Huber 1993; Fernández and Sharkey 2006). To identify genera within the corresponding superfamilies and families, we consulted multiple taxonomic keys (Masner 1976; Hayat 1983; Gibson 1995; Gibson et al. 1997; Heraty 2002; Trjapitzin et al. 2008; Pezzini and Köhler 2017; Buffington et al. 2020; Huber et al. 2021). Morphospecies is a useful approach when detailed taxonomic information is limited (Derraik et al. 2010). For specimens that could not be identified to species, these were identified to the lowest taxonomic level reached, usually genus and in some cases family, and defined as morphospecies using combinations of morphological characters that were consistent across multiple specimens. Whenever available, we used characters included in taxonomic keys. We also compared our specimens with reference specimens assigned to previously established morphospecies at the University of California, Riverside collection (**UCRC**) to maintain consistency and avoid duplicate morphospecies assignments. Morphospecies assignments were also discussed with specialists familiar with the taxonomic groups. For Formicidae, species identifications followed published keys (Bolton 1994; Herrera et al. 2024). For all identified species, we conducted a literature review looking for previous records in the archipelago, establishment status in Galápagos (endemic, native, introduced, cryptogenic, or undetermined), and taxonomic status.

### Dataset integration

The four source datasets were integrated into a single dataset using the R packages ‘dplyr’ (Wickham et al. 2025), ‘tidyr’ (Wickham et al. 2024), and ‘stringr’ (Wickham 2025) in R version 4.4.1 (R Core Team 2024). All dataset fields were standardized following the Darwin Core standard (DwC; Wieczorek et al. 2012). During this process, the lowest taxonomic level available, whether superfamily, family, genus, or species, was used. We applied data-cleaning techniques to unify taxonomic nomenclature and geographic information (Suppl. material 3: fig. S2). For records identified as species/morphospecies that lacked geographic coordinates, approximate coordinates were assigned based on the available locality information. Records that reported only the island were assigned the geometric centroid of that island’s terrestrial polygon, whereas records that included a locality within an island were assigned a coordinate located within or near the indicated locality. Records georeferenced through this procedure were identified in the integrated dataset (Suppl. material 2).

Information on vegetation zones was standardized following the classification described for the Galápagos Islands by Jackson (2006), using the categories littoral, dry, transition, and humid (Fig. 1, Suppl. material 3: fig. S1A–D).

Each taxon was categorized as endemic, native, introduced, cryptogenic, or undetermined (Suppl. material 1). Endemic taxa are those found only in the Galápagos Islands; native taxa are also found elsewhere and are considered to have dispersed naturally to Galápagos, whereas introduced taxa arrived through human activities (Sinclair 2023). In this study, the cryptogenic category was used only when the origin remained unresolved after sufficient research or review. Undetermined taxa are those whose origin had not been evaluated or could not be evaluated because the available information or taxonomic resolution was insufficient.

### Statistical analyses

The number of superfamilies, families, genera, and species/morphospecies, as well as the proportion of unique and shared species/morphospecies, were calculated for each island and vegetation zone. Additionally, we analyzed the relationship between island area, as reported by Snell et al. (1996), and species/morphospecies richness. Due to the extreme variance in island size, which spans several orders of magnitude (e.g., Isabela vs Darwin), and the discrete nature of the species data, standard parametric assumptions were violated. High-leverage outliers can severely distort parametric generalized linear models by inflating standard errors (Zar 2010). Therefore, to study this relationship while accounting for uneven sampling effort, we conducted a nonparametric Spearman partial correlation analysis (r_s_). Sampling effort was represented by the number of occurrence records per island after filtering the integrated dataset to records identified as species, subspecies, or morphospecies. Rank-based partial correlation is robust against non-normal distributions and extreme outliers (Bishara and Hittner 2012). To account for right-skewed data and non-linear power-law scaling, we log-transformed island area (ha) and sample size (*n*). Even though there are alternatives to occurrence records to explore the area/species relationship, those require a more uniform sampling structure which was not achievable in the context of this study which integrated several data sources along 65 years. In addition, restricting the tests to each type of method will reduce the heuristic value of the data and will not allow an understanding of the diversity of the order as several traps are strongly biased taxonomically. The analysis was performed using the ppcor package (Kim 2015) and plotted in a scatter plot for visualization (Fig. 3).

To estimate overall biodiversity patterns across the archipelago, we analyzed species richness using georeferenced occurrence records assigned to species/morphospecies (*n* = 19,355). We divided the geographic extent of all islands into a 0.1° × 0.1° grid. For each quadrant of the grid, species/morphospecies richness and the number of occurrence records were calculated using the rasterize function from the R package raster (Hijmans 2018). Only grid cells with at least one record were included, resulting in 93 occupied cells. We constructed a binary presence–absence matrix consisting of grid cells as rows and species/morphospecies as columns. This matrix was used to generate a sample-based species accumulation curve and calculate the incidence-based richness estimators Chao2, Jackknife 1, and Bootstrap with 100 permutations using the poolaccum function from the R package vegan (Oksanen et al. 2001). Individual-based rarefaction was not used because individual counts and sampling effort were not standardized or directly comparable among the heterogeneous data sources included in the integrated dataset.

A non-metric multidimensional scaling (NMDS) analysis was performed to assess differences in species composition among islands using records from the integrated dataset with complete information for island, vegetation zone, and species/morphospecies identity (*n* = 19,177). NMDS was performed using the metaMDS function from the R package vegan (Oksanen et al. 2001), specifying two dimensions (k = 2), with a maximum of 999 iterations and 500 random starts to avoid local minima and ensure convergence. A presence–absence matrix with species/morphospecies as columns and each combination of island and vegetation zone as rows was used to calculate Jaccard dissimilarity. For visualization, 95% confidence ellipses were drawn for groups with sufficient observations.

All graphs were generated using the ggplot2 package (Wickham 2016) in R. Maps were generated using the raster and sf packages (Pebesma 2018), and some color palettes were derived from the viridis package (Garnier et al. 2023). The Figshare repository contains all data and R scripts required to reproduce the statistical analyses and figures. The repository is organized as an RStudio Project to provide a consistent working directory and facilitate reproducible execution. Package versions and dependencies are managed using the renv package (Ushey and Wickham 2025), ensuring a reproducible computational environment.

## Results

### Survey effort and richness

**Figure 2.**
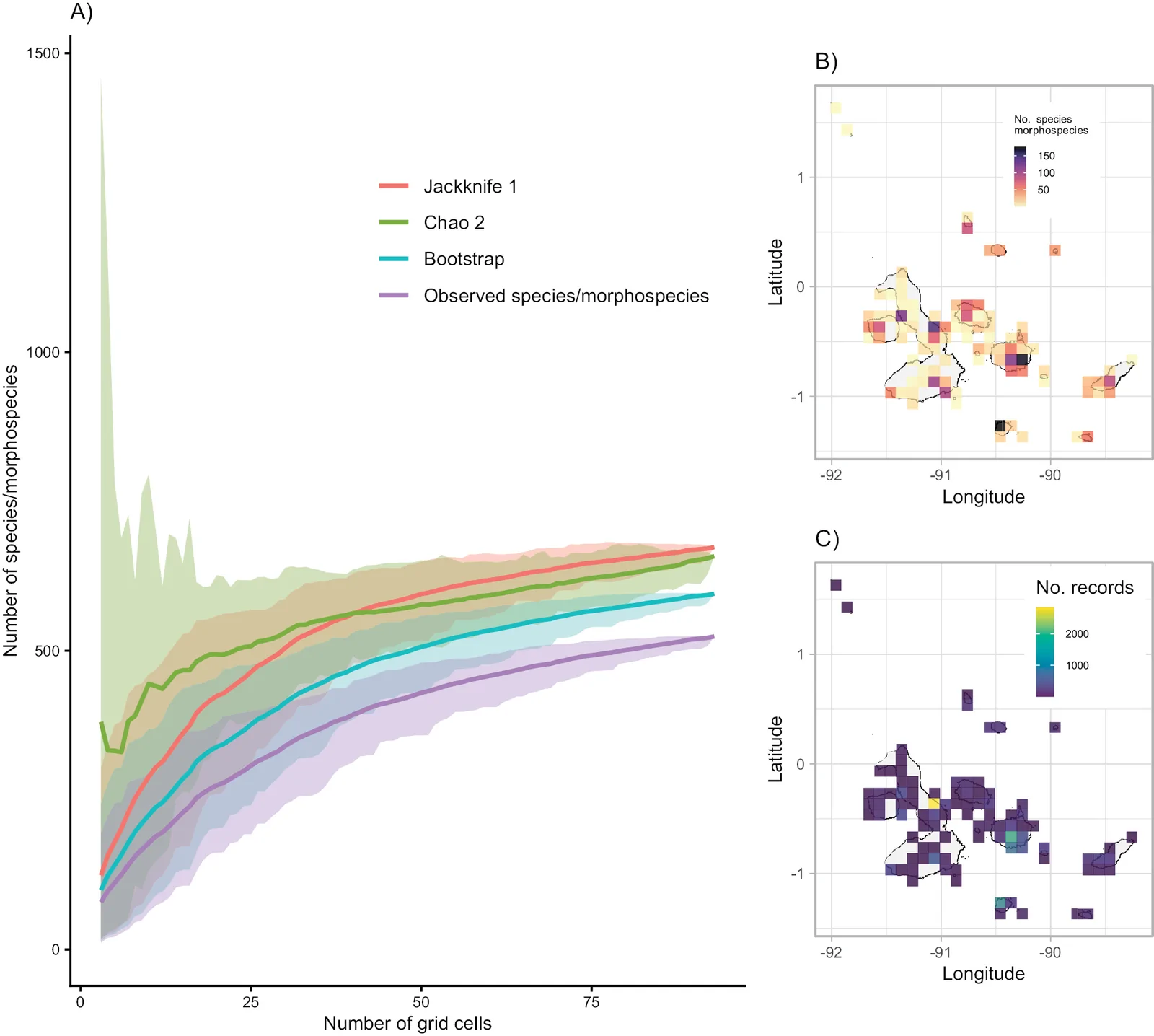
Spatial accumulation and distribution of documented Hymenoptera diversity across the Galápagos archipelago. **A**. Sample-based accumulation curve and incidence-based richness estimators (Jackknife 1, Chao2, and Bootstrap) across 93 occupied 0.1° × 0.1° grid cells; shaded bands represent 95% confidence intervals; **B**. Richness of species/morphospecies per grid cell; **C**. Number of occurrence records per grid cell.

Overall, the integrated dataset included 21,364 records of Hymenoptera belonging to 12 superfamilies, 44 families, 82 subfamilies, 222 genera, 137 species, 1 subspecies, and 386 morphospecies (for a total of 524 species/morphospecies). Our study provides new records of 20 species and 134 genera for the archipelago, representing 11 families not previously recorded from Galápagos (Suppl. material 1). The species/morphospecies accumulation curve and incidence-based richness estimators yielded values above the observed richness (Bootstrap = 595.47, Jackknife 1 = 673.38, Chao2 = 658.26; Fig. 2), indicating that, despite the substantial increase in documented diversity, additional sampling and taxonomic work may document further species/morphospecies. Records discriminated by year show a small increase around the late 1990s and early 2000s (Peck et al. 1998; Tye et al. 2002), but the largest increase in documented species/morphospecies occurred during the current study (Suppl. material 3: fig. S2A).

The sampling methods recorded in the integrated dataset (Suppl. material 2) yielded the following data: pitfall traps accounted for 6,677 records, followed by Malaise traps (2,481), baits (1,887), aspirators (1,810), pan traps (1,336), manual collection (1,202), Winkler extraction (492), and sweep nets (337). For 394 species/morphospecies, the sampling method was indicated. Of these, 174 (44.2%) were recorded exclusively by a single method. Malaise traps contributed the highest number of method exclusive species/morphospecies (129), followed by pan traps (24), sweep nets (10), manual collection (5), pitfall and Berlese traps (2 each), and aspirators and Winkler extraction (1 each).

### Taxonomic diversity, establishment status, and distribution

**Figure 3.**
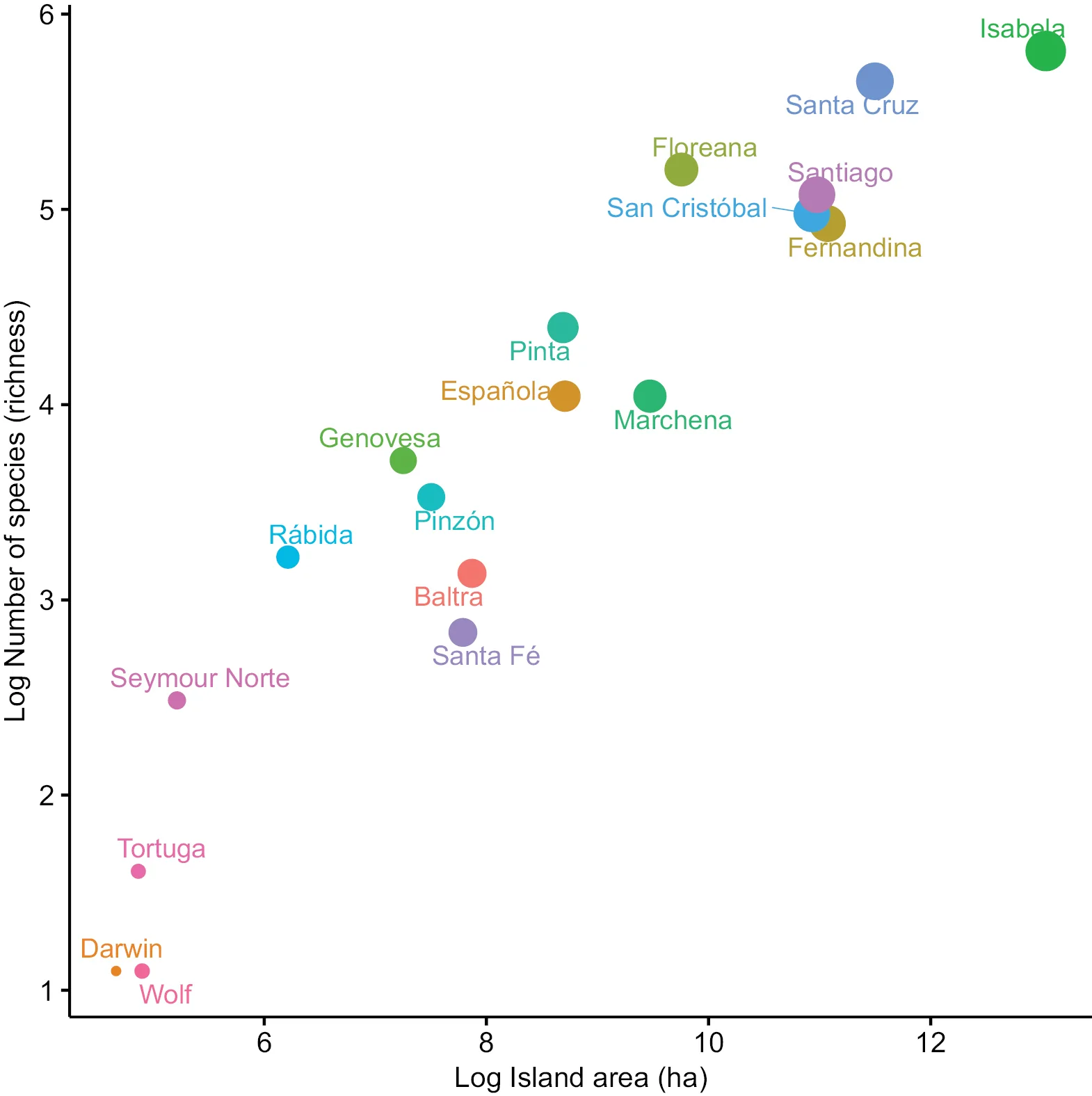
Relationship between island area and Hymenoptera species/morphospecies richness across 18 islands of the Galápagos archipelago. Island area and species/morphospecies richness were log-transformed for visualization. Point size represents sampling effort, measured as the number of species/morphospecies occurrence records per island. The partial Spearman correlation indicated a positive association after accounting for sampling effort (r_s_ = 0.63, p = 0.006, *n* = 18).

At the superfamily level, the most species-rich groups were Chalcidoidea, Ichneumonoidea, Vespoidea, Chrysidoidea, and Platygastroidea (Suppl. material 3: fig. S3). At the family level, were Encyrtidae, Eulophidae, Formicidae, Scelionidae, and Braconidae (Suppl. material 3: fig. S4). Of the 524 species/morphospecies documented, 452 (86.3%) were assigned to taxonomic groups with predominantly parasitoid biology, including 82 species and 370 morphospecies. Formicidae accounted for 53 (10.1%), including 43 species, one subspecies, and nine morphospecies; Apoidea for 16 (3.1%), including nine species and seven morphospecies; and Vespidae for three (0.6%), all identified to species level. Among the 137 named species, 30 (21.9%) were classified as endemic and represented nine families, with the highest numbers recorded in Dryinidae (9 species), Formicidae (8), and Ichneumonidae (5). Five species (3.6%) were classified as native, two (1.5%) as cryptogenic, and 39 (28.5%) as undetermined, whereas 61 (44.5%) were introduced (Suppl. material 1). Higher observed species/morphospecies richness was recorded on Isabela, Santa Cruz, Floreana, Santiago, San Cristóbal, and Fernandina while lower richness was recorded on Wolf, Darwin, Tortuga, and Seymour Norte (Fig. 3). The partial Spearman correlation indicated a positive association between island area and species/morphospecies richness after accounting for sampling effort, measured as the number of species/morphospecies occurrence records per island (r_s_ = 0.63, p = 0.006, *n* = 18; Fig. 3).

**Figure 4.**
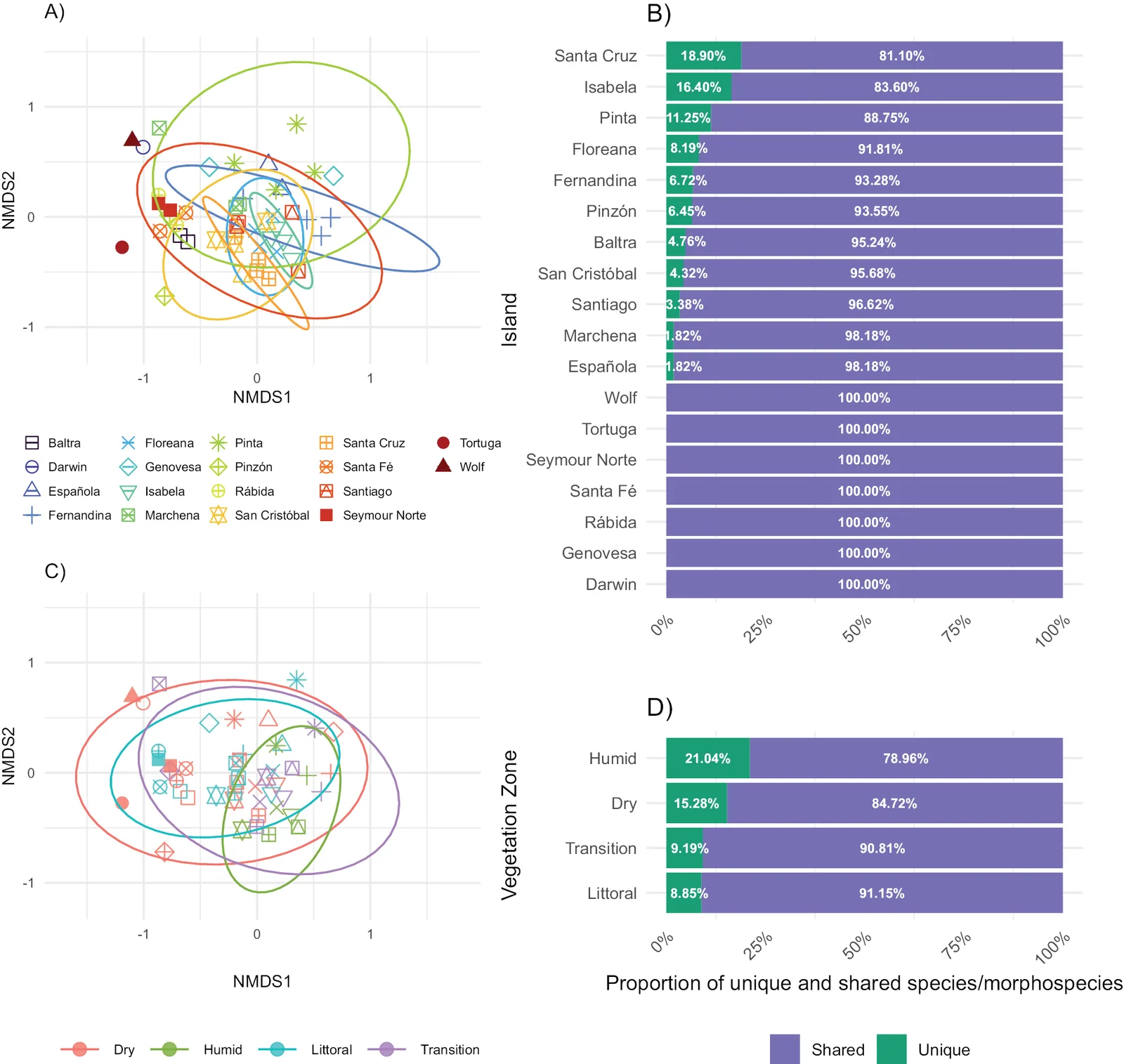
Non-metric multidimensional scaling (NMDS) ordinations and percentages of unique and shared species/morphospecies among islands and vegetation zones of the Galápagos archipelago. The NMDS ordinations were based on a Jaccard dissimilarity matrix of Hymenoptera species/morphospecies composition, with a stress of 0.12. **A**. NMDS ordination by island; **B**. Percentages of unique and shared species/morphospecies by island; **C**. NMDS ordination by vegetation zone; **D**. Percentages of unique and shared species/morphospecies by vegetation zone. Ellipses in panels **A** and **C** represent 95% confidence ellipses.

Most of the species/morphospecies recorded on each island were also recorded on at least one other island, with shared proportions ranging from 81.1% on Santa Cruz to 100% on seven islands (Fig. 4B). The islands with the highest proportions of unique species/morphospecies were Santa Cruz (18.9%), Isabela (16.4%), and Pinta (11.3%), whereas Darwin, Genovesa, Rábida, Santa Fe, Seymour Norte, Tortuga, and Wolf had no unique species/morphospecies (Fig. 4B). In the NMDS ordination (stress = 0.12), several islands showed overlap in their species/morphospecies composition (Fig. 4A). The Jaccard-based heatmap and dendrogram also showed that dissimilarity varied among islands (Suppl. material 3: fig. S5).

A similar pattern was observed among vegetation zones, where most species/morphospecies were shared, with shared proportions ranging from 79% in the humid zone to 91.2% in the littoral zone (Fig. 4D). The humid zone had the highest proportion of unique species/morphospecies (21%), followed by the dry zone (15.3%), the transition zone (9.2%), and the littoral zone (8.9%) (Fig. 4D). The vegetation zones showed overlap in the NMDS ordination, with greater overlap among the dry, littoral, and transition zones (Fig. 4C). In the dendrogram based on Jaccard dissimilarity, the dry and transition zones clustered first, followed by the littoral zone, whereas the humid zone joined the cluster at the highest dissimilarity (Suppl. material 3: fig. S6).

### Spatial distribution

Our study provides new distributional information for Hymenoptera across multiple islands and localities, as reflected by the spatial patterns of species/morphospecies richness and occurrence records across the archipelago (Figs 1, 2B, C). Higher concentrations of observed richness and occurrence records were found in several areas of Isabela, Santa Cruz, Floreana, Santiago, and San Cristóbal. Areas with higher observed richness partly coincided with areas containing a greater number of occurrence records.

**Figure 5.**
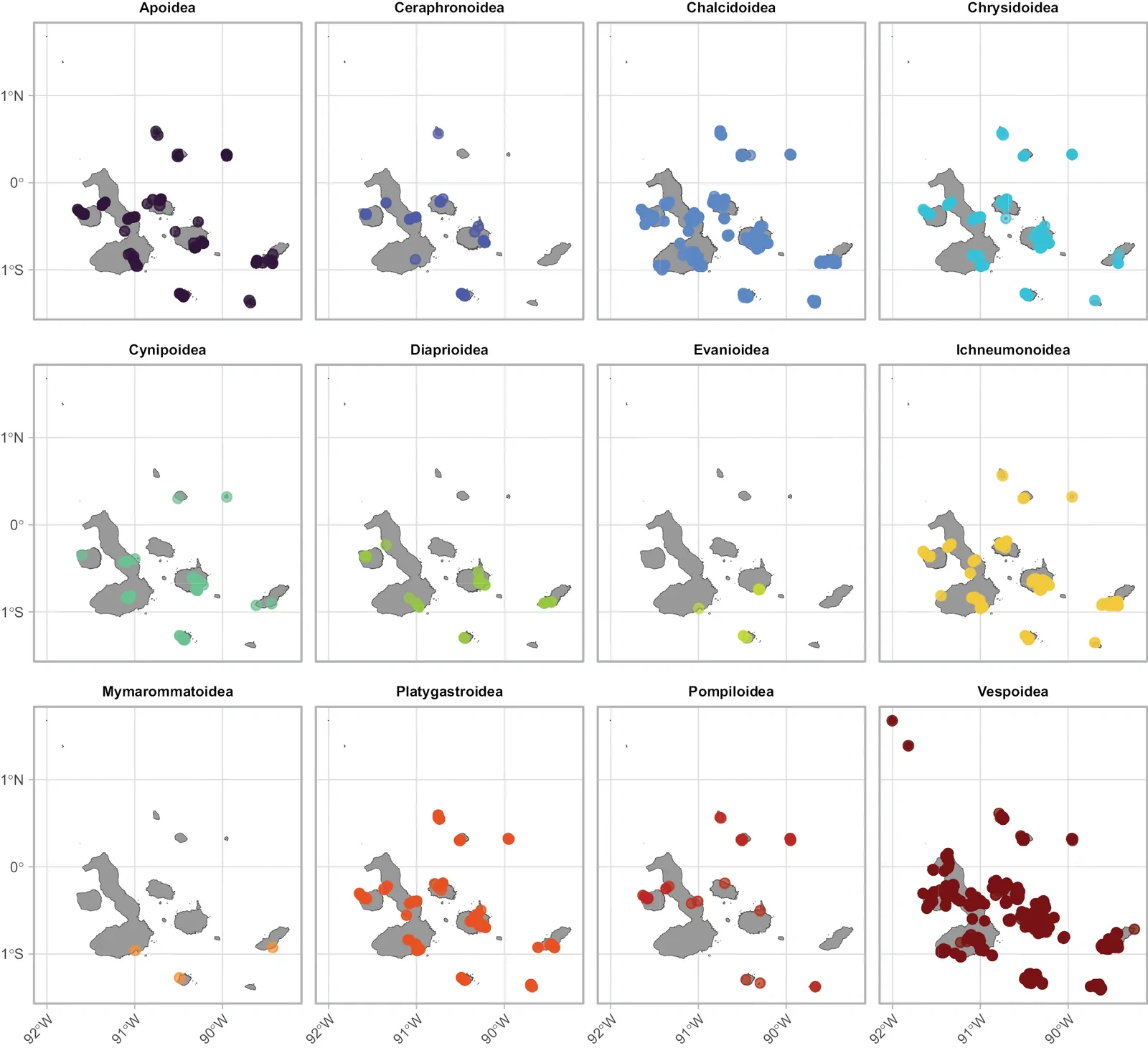
Geographic distribution of Hymenoptera superfamilies across the Galápagos Islands based on all sources of information. Country boundaries were obtained from Natural Earth (Admin 0 – Countries, naturalearthdata.com). Map projected in WGS 84 / UTM Zone 15S.

Hymenopteran superfamilies exhibited distinct distribution patterns across the Galápagos Islands. Vespoidea showed the broadest latitudinal (3.09°) and longitudinal (2.76°) ranges and occupied the most islands (18). Apoidea, Chalcidoidea, Chrysidoidea, Cynipoidea, Ichneumonoidea, Platygastroidea, and Pompiloidea displayed widespread distributions without evident geographic patterns. Ceraphronoidea was absent from the eastern islands (San Cristóbal and Española), whereas Diaprioidea was not recorded from the northern islands (Pinta, Genovesa, and Marchena). Evanioidea (0.561° latitudinal range) and Mymarommatoidea (0.347° latitudinal range) showed the most restricted distributions, limited to southern islands (Floreana, south of Isabela and San Cristóbal) (Fig. 5). At the family level, distribution patterns generally coincided with those observed in the superfamilies. Several families, such as Formicidae, Braconidae, Encyrtidae, Eulophidae, Pteromalidae, and Vespidae, were present on numerous islands. In contrast, families such as Eurytomidae, Evaniidae, Mymarommatidae, Platygastridae, and Sphecidae were recorded from fewer islands (Suppl. material 3: fig. S7).

## Discussion

We report Hymenoptera as one of the most species-rich insect orders in the Galápagos Islands; with 524 species/morphospecies this is numerically higher than the 486 species reported for Coleoptera (Peck 2006), although the Hymenoptera total includes morphospecies and the two inventories differ in taxonomic resolution. Previous studies on Galápagos biodiversity mention lower numbers for Hymenoptera and in some cases do not provide an associated list (Linsley and Usinger 1966; Peck et al. 1998). The most recent work prior to this study provided a list of 84 species, which was the most complete Hymenoptera inventory available at that time (Heraty and Herrera 2017). Our results substantially increase the documented number of species/morphospecies in the islands with a detailed list to establish a clear taxonomic baseline. This study highlights the need for comprehensive taxonomic inventories even for hyperdiverse groups in an intensively studied region with unique biodiversity, such as the Galápagos.

Despite the broad compilation of museum, literature, and online records, together with the structured sampling component of the wasp dataset, additional diversity may remain undocumented for several reasons. First, the incidence-based Bootstrap, Jackknife 1, and Chao2 estimators yielded richness values above the observed number of species/morphospecies. Second, most morphospecies were assigned to taxonomic groups with predominantly parasitoid biology, which contain substantial undescribed diversity worldwide (Forbes et al. 2018). Third, studies in island systems such as Madagascar and Hawaii have reported that parasitoid diversity is related to host availability (Whitfield 1998; Forbes et al. 2018), and there is a high number of potential host species in the Galápagos archipelago as records of Coleoptera, Diptera, and Lepidoptera total 1,121 species (Peck 2006; Roque-Albelo and Landry 2016; Sinclair 2023). Although morphospecies allow diversity patterns to be documented, continued detailed taxonomic work is needed to clarify their species-level identities and improve our understanding of hymenopteran diversity in the archipelago.

### Hymenoptera richness across islands

Overall, the partial Spearman correlation indicated a positive association between island area and species/morphospecies richness after accounting for sampling effort, a pattern broadly consistent with island biogeographic expectations regarding the influence of island size on species richness (MacArthur and Wilson 2001; Whittaker and Fernández-Palacios 2007). Conversely, smaller islands such as Wolf, Darwin, Tortuga, and Seymour Norte showed lower observed species/morphospecies richness (Fig. 3). The positive relationship between island area and richness has been documented in other insect groups in the Galápagos. For example, in both Coleoptera and Lepidoptera, species richness correlates with island area (Peck 2006; Roque-Albelo 2006). In Coleoptera, richness of native species not only increases with island area, but with elevation as well (Peck 2006). The observed area–richness relationship may be the result of larger islands providing more suitable habitats, as similar results have been found in other archipelagos and with other taxonomic groups (Mühlenberg et al. 1977; Wardle et al. 1997; Lomolino and Weiser 2001).

### Species composition between islands and vegetation zones

We found that most species/morphospecies were shared among islands and vegetation zones (i.e., seven islands had no unique species/morphospecies, whereas the highest proportions of unique species/morphospecies were recorded on Santa Cruz, Isabela, and Pinta; Fig. 4B). Previous studies of other insect groups in Galápagos have focused on documented taxonomic diversity, species distributions, and origin status rather than quantitative comparisons of compositional similarity across large lineages (Peck 2006; Roque-Albelo and Landry 2016; Sinclair 2023). Therefore, our study provides the first detailed assessment for a large order such as Hymenoptera and shows that most species/morphospecies were shared among islands. Comparable analyses of other insect orders would help determine whether the high proportion of shared species/morphospecies observed here is a broader pattern across the archipelago. The humid zone showed the highest proportion of unique species/morphospecies (21%) (Fig. 4D). The higher proportion of unique species/morphospecies in the humid zone is consistent with previous observations (Peck 2001). Similarly, some lepidopteran and spider species are restricted to lowland or highland habitats (Roque-Albelo 2006; Baert 2024). Such patterns may be associated with differences in mobility (Peck 2001; Baert 2024). The compositional overlap observed among some islands may partly reflect aerial dispersal, which has been documented for insects, including Hymenoptera, between islands in the Galápagos archipelago (Peck 1994; Picón-Rentería et al. 2024).

### Introduced hymenopterans in the Galápagos

The proportion of introduced taxa (44.5% of the species; Suppl. material 1) is higher than the 23% of insects estimated previously (Causton et al. 2006), the 17.5% (588 out of ~3,360 species) of the non-hymenopteran invertebrates, and the 20.7% (540 of ~2,615 species) of the non-hymenopteran insects based on the databases from the Charles Darwin Foundation (Charles Darwin Foundation 2025a, 2025b). Even though there are several studies showing an increased number and expansion of introduced species in the archipelago (Toral-Granda et al. 2017; Herrera et al. 2024; Picón-Rentería et al. 2024), our results may be influenced by the exclusion of taxa with undetermined establishment status. Taxonomic studies integrating multiple data sources can clarify the biogeographic affinities between Hymenoptera from the Galápagos and the South American mainland and thus help to understand the origin of many Galápagos hymenopterans.

### Undocumented diversity and taxonomic gaps

Of the 452 species and morphospecies assigned to taxonomic groups with predominantly parasitoid biology, 370 were morphospecies, indicating that most of the unresolved taxonomic diversity documented in this study is concentrated in parasitoid Hymenoptera. Globally, parasitoid wasps are highly diverse but remain relatively poorly known; their small body size, historical collecting biases, and the relative lack of taxonomic resources have contributed to their understudy (Forbes et al. 2018). Our results highlight parasitoid Hymenoptera as one of the main priorities for taxonomic resolution to advance the inventory of Galápagos Hymenoptera. Continued taxonomic work on this group, together with the study of its host associations, is necessary to clarify the identities of these morphospecies and increase knowledge of the diversity, establishment status, and distribution of Hymenoptera in the archipelago.

### Island biogeography and species richness

In many cases, the postulates of island biogeography apply directly to species evolution and distributional patterns in the Galápagos (Losos and Ricklefs 2009; Grant 2017). For instance, isolation, dispersal ability, and habitat specialization have influenced colonization and diversification among Galápagos terrestrial lineages (Parent et al. 2008). Similarly, within-island factors such as area and vegetation-zone variation have been associated with the richness of mammals and low-dispersing invertebrates (Schenk and Steppan 2018; Roell et al. 2021). *Naesiotus* land snails (Phillips et al. 2020), *Hogna* spiders (De Busschere et al. 2010), and *Stomion* and *Galapaganus* beetles (Sequeira et al. 2008) are examples of the island progression rule and *in situ* speciation events (MacArthur and Wilson 1963). In addition to these colonization and speciation patterns, island area is one of the central factors considered in island biogeography theory and may influence species richness. For Hymenoptera, the partial Spearman correlation indicated a positive association between island area and species/morphospecies richness after accounting for sampling effort. Larger islands might provide more habitats supporting a wider range of species (Tye et al. 2002). However, human activities may also modify these patterns by facilitating the introduction and spread of non-native species (Toral-Granda et al. 2017), with some islands experiencing important human settlements that dated back over a century such as San Cristóbal and Santa Cruz (Stahl et al. 2020). Studies of vespids and parasitoid wasps have also highlighted the role of human activities in the introduction of Hymenoptera (Camacho-Erazo et al. 2024; Camargo-Martinez et al. 2024; Picón-Rentería et al. 2024; Sandoval-B et al. 2024). The number of introduced Hymenoptera recorded may also be influenced by parasitoids being transported with their introduced hosts (Camacho-Erazo et al. 2024).

## Conclusions

Structured and non-structured surveys and data from multiple sources provide a powerful baseline of information to better characterize the biodiversity of a region. For a given area, this work provides grounded results on documented richness, the extent of endemism, and geographic distribution patterns. We document a notable increase in Hymenoptera richness with an explicit list of species/morphospecies, placing the order among the most species-rich insect orders documented in the archipelago. Although the Galápagos archipelago is well known for its endemic taxa, our results indicate that introduced species currently represent a substantial proportion of the named Hymenoptera recorded. However, this proportion is based only on named species and may change as the remaining morphospecies are formally identified. Detailed taxonomic studies will be necessary to provide a more complete assessment.

The 20 species and 134 genera newly recorded for Galápagos in this study, representing 11 previously unrecorded families, demonstrate how much of the hymenopteran diversity of the archipelago remained undocumented. Together with the 386 morphospecies documented and richness estimates above the observed richness, these results show that much of this historically understudied diversity remains unresolved. Much of this unresolved diversity is concentrated in parasitoid lineages, highlighting them as a priority for continued taxonomic work. Additional sampling and taxonomic study may document further species/morphospecies and increase the known richness of Hymenoptera in the archipelago.

Our findings enrich our understanding of Hymenoptera diversity and distribution in the Galápagos, providing an essential data baseline for future research and conservation efforts. We are aware that our taxonomic characterization relies primarily on morphological evidence; therefore, integrative taxonomic approaches combining morphological and molecular evidence will be important for identifying many of the morphospecies recorded. We suggest that the taxonomic resolution of these Galápagos morphospecies may also benefit from integrative taxonomic studies of related Hymenoptera from the South American mainland.

## Additional information

### Artificial Intelligence (AI) use

The authors accept full responsibility for the content of the manuscript, including the disclosure of any use of AI.

No AI tools were used in the preparation of this manuscript.

### Author ORCIDs

P. Picón-Rentería https://orcid.org/0009-0008-5341-4094

H.W. Herrera https://orcid.org/0000-0002-1417-4349

J.M. Avendaño https://orcid.org/0009-0003-2570-7707

M.C. Tocora-Protz https://orcid.org/0000-0002-5304-4010

A.F. Sánchez Restrepo https://orcid.org/0000-0001-7514-068X

F. Fernández https://orcid.org/0000-0002-6862-3592

J.M. Heraty https://orcid.org/0000-0002-9246-5651

A.Y. Bui https://orcid.org/0009-0008-0023-3759

S. Peck https://orcid.org/0000-0002-3407-4978

C.E. Sarmiento https://orcid.org/0000-0003-4012-8108

### Data availability

All data and code supporting this study are openly available in the following Figshare repository: https://doi.org/10.6084/m9.figshare.32050638. The file cited in the manuscript as Suppl. material 1 is provided under the name “Table_S1_Taxonomic_checklist.xlsx”, whereas the integrated dataset cited as Suppl. material 2 is provided as “Table_S2_Hymenoptera_Galapagos_integrated dataset.csv”. Both files accompany the article as supplementary material and are also archived in the Figshare repository under the same file names. The repository also contains the R project and scripts necessary to reproduce the analyses and figures presented in this study, together with additional information in the readme.md file.

## Supplementary materials

Supplementary material 1 Taxonomic checklist of Hymenoptera reported from the Galápagos Archipelago Data type xlsx Explanation note Provides a summarized taxonomic checklist of Hymenoptera reported from the Galápagos Archipelago. It includes taxonomic information, taxon rank, new-record status for Galápagos, establishment means, source dataset, and number of records for each species, subspecies, and morphospecies. https://binary.pensoft.net/file/1805366This dataset is made available under the Open Database License (http://opendatacommons.org/licenses/odbl/1.0/). The Open Database License (ODbL) is a license agreement intended to allow users to freely share, modify, and use this Dataset while maintaining this same freedom for others, provided that the original source and author(s) are credited. Patricio Picón-Rentería, Henri W. Herrera, José M. Avendaño, María C. Tocora-Protz, Andrés F. Sánchez Restrepo, Fernando Fernández, John M. Heraty, Ashley Y. Bui, Stewart Peck, Carlos E. Sarmiento

Supplementary material 2 Consolidated database of Hymenoptera occurrence records from the Galápagos Archipelago Data type csv Explanation note Contains the consolidated database integrating the four source datasets: wasp dataset, ant dataset, museum dataset, and literature dataset. It includes 21,364 occurrence records with taxonomic, geographic, temporal, locality, sampling-method, source-dataset, and establishment-status information. https://binary.pensoft.net/file/1805367This dataset is made available under the Open Database License (http://opendatacommons.org/licenses/odbl/1.0/). The Open Database License (ODbL) is a license agreement intended to allow users to freely share, modify, and use this Dataset while maintaining this same freedom for others, provided that the original source and author(s) are credited. Patricio Picón-Rentería, Henri W. Herrera, José M. Avendaño, María C. Tocora-Protz, Andrés F. Sánchez Restrepo, Fernando Fernández, John M. Heraty, Ashley Y. Bui, Stewart Peck, Carlos E. Sarmiento

Supplementary material 3 Supplementary images Data type pdf https://binary.pensoft.net/file/1805368This dataset is made available under the Open Database License (http://opendatacommons.org/licenses/odbl/1.0/). The Open Database License (ODbL) is a license agreement intended to allow users to freely share, modify, and use this Dataset while maintaining this same freedom for others, provided that the original source and author(s) are credited. Patricio Picón-Rentería, Henri W. Herrera, José M. Avendaño, María C. Tocora-Protz, Andrés F. Sánchez Restrepo, Fernando Fernández, John M. Heraty, Ashley Y. Bui, Stewart Peck, Carlos E. Sarmiento
