# Supplementary material for: Hymenoptera of Galápagos: diversity and distribution of one of the most species-rich insect orders in the archipelago
Source: Zookeys. 2026 Oct 9;1294:229–51. doi: 10.3897/zookeys.1294.200001 (PMC13675722; doi:10.3897/zookeys.1294.200001)
Supplement: Supplementary file 1 [file zookeys-1294-229_article-200001__-s003.pdf]

Supplementary material

Hymenoptera of Galápagos: diversity and distribution of one of the most species-rich insect orders in the archipelago

Patricio Picón-Rentería*, Henri W. Herrera, José M. Avendaño, María C. Tocora-Protz, Andrés F. Sánchez Restrepo, Fernando Fernández, John M. Heraty, Ashley Y. Bui, Stewart Peck, and Carlos E. Sarmiento*

Patricio Picón-Rentería; Carlos E. Sarmiento

Email: rpicon@unal.edu.co; cesarmientom@unal.edu.co

This PDF file includes:

Figures S1 to S7

Data Accessibility: All data and code supporting this study are openly available in the following Figshare repository: https://doi.org/10.6084/m9.figshare.32050638. The file cited in the manuscript as Suppl. material: Table S1 is provided under the name "Table_S1_Taxonomic_checklist.xlsx", whereas the integrated occurrence dataset cited as Suppl. material: Table S2 is provided as "Table_S2_Hymenoptera_Galapagos_integrated dataset.csv". Both files accompany the article as supplementary material and are also archived in the Figshare repository under the same file names. The repository also contains the R project and scripts necessary to reproduce the analyses and figures presented in this study, together with additional information in the readme.md file.

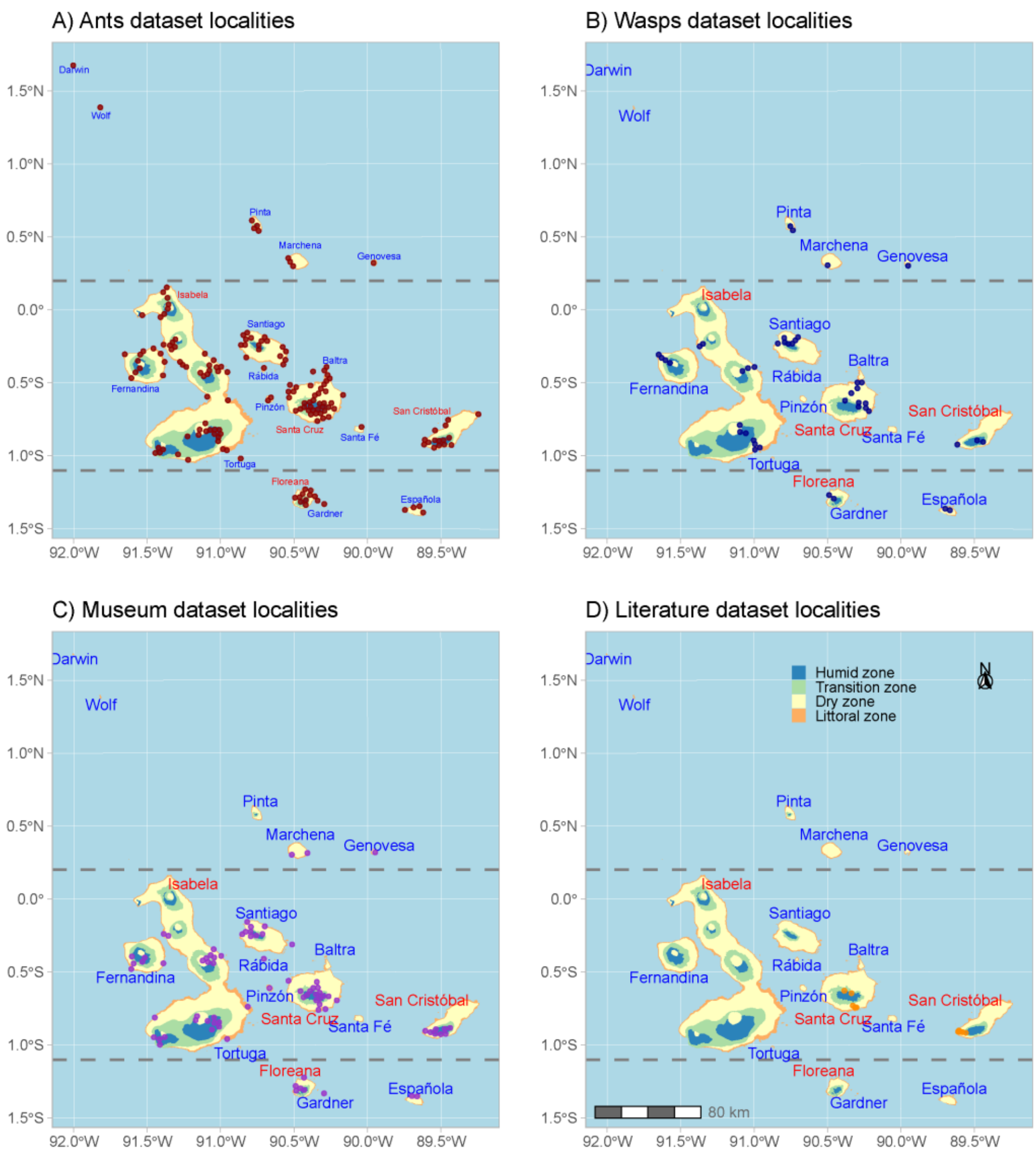

Figure S1. Distribution of the localities represented by records with geographic coordinates in the four datasets used in this study: wasp dataset, ant dataset, museum dataset, and literature dataset. Island names shown in red indicate inhabited islands. Vegetation zone classification follows previously described schemes (Jackson 2006). Country boundaries were obtained from Natural Earth (Admin 0 – Countries, naturalearthdata.com). Maps projected in WGS 84 / UTM Zone 15S.

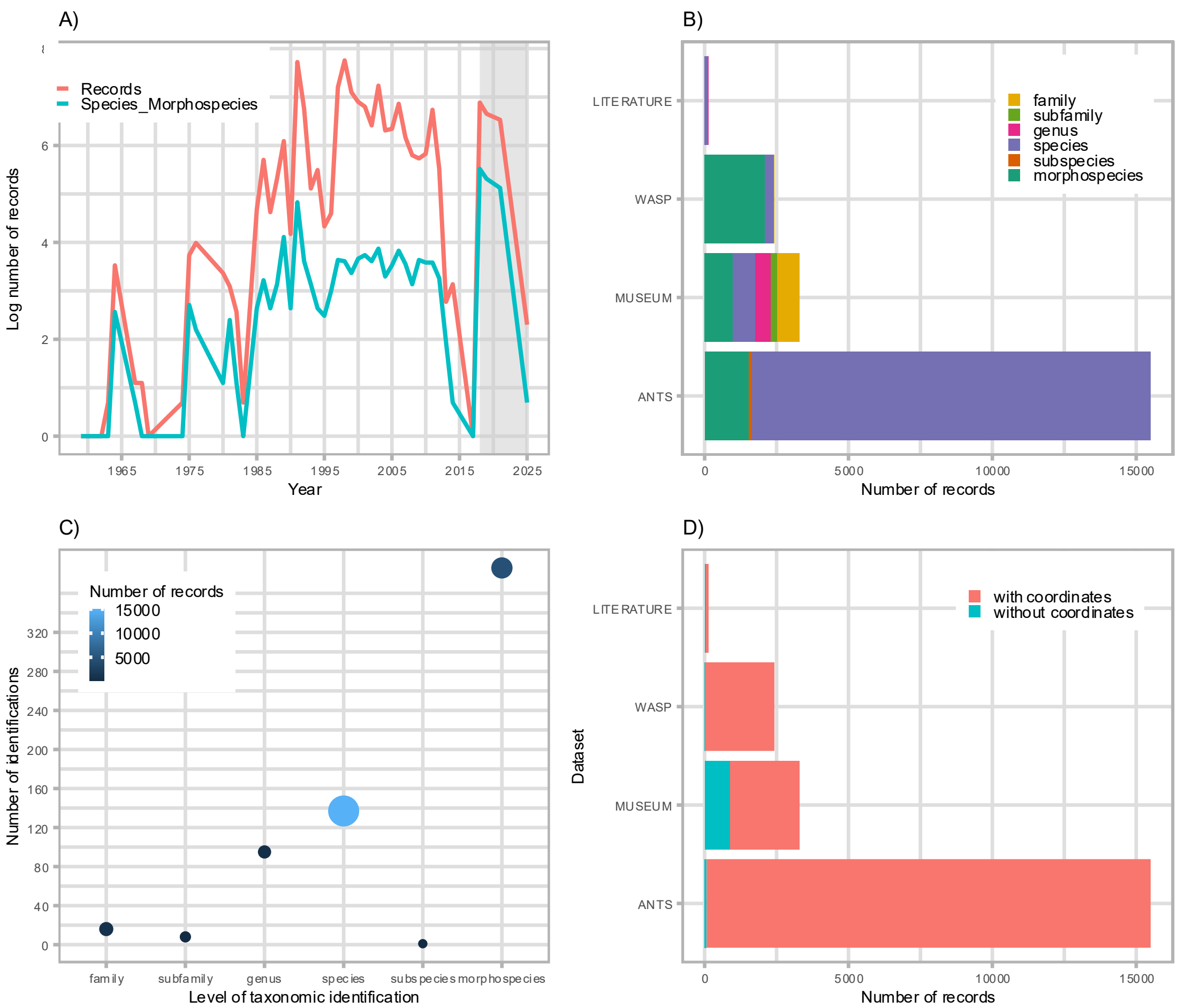

Figure S2. Temporal, taxonomic, and geographic coverage of the four source datasets. A. Log-transformed number of records and species/morphospecies recorded per year; the gray-shaded area indicates the period represented by the wasp dataset, including structured sampling conducted in 2018, 2019, and 2021 and opportunistic records from 2025. B. Level of taxonomic identification across the different datasets. C. Number of identifications and records by taxonomic level. D. Number of records with and without coordinates per dataset.

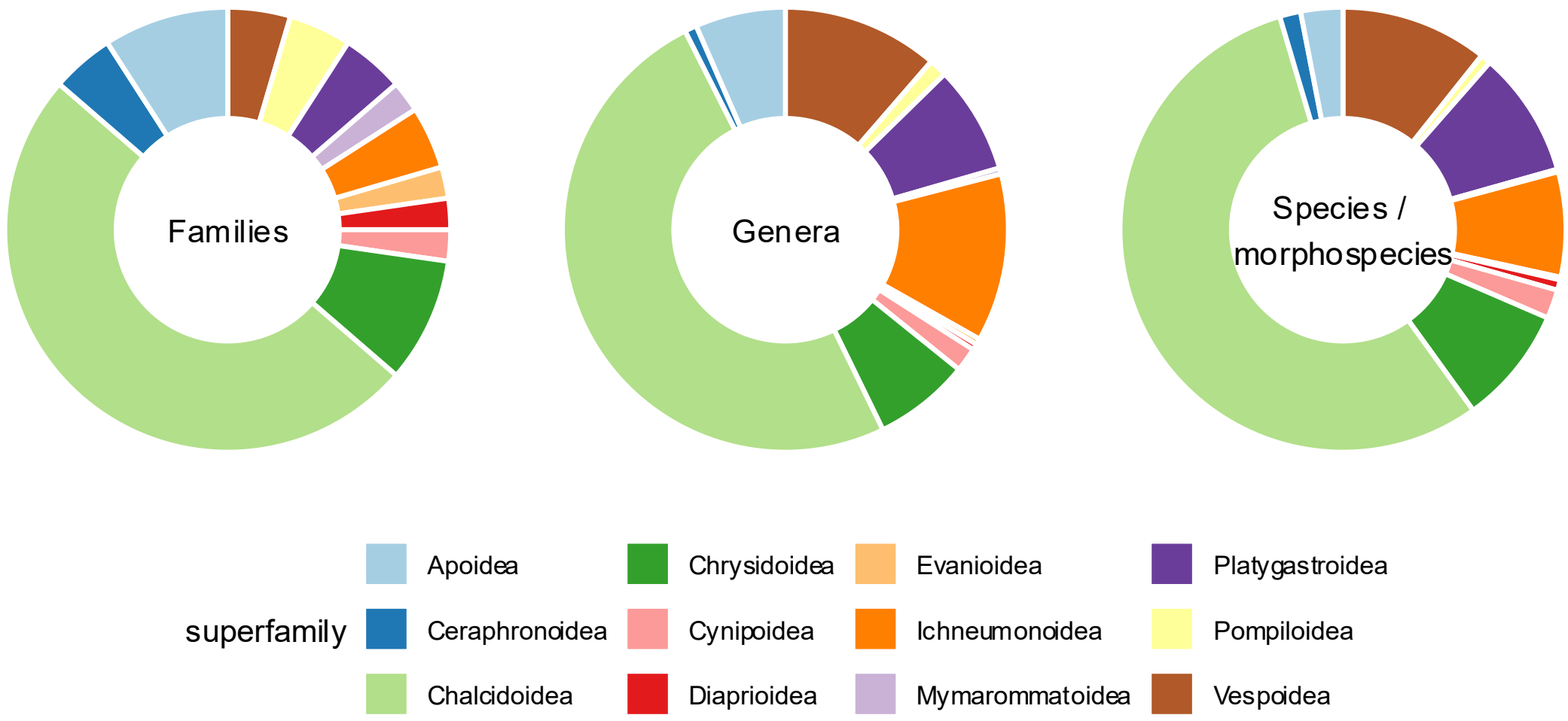

Figure S3. Proportional representation of Hymenoptera superfamilies among the families, genera, and species/morphospecies documented in the integrated dataset.

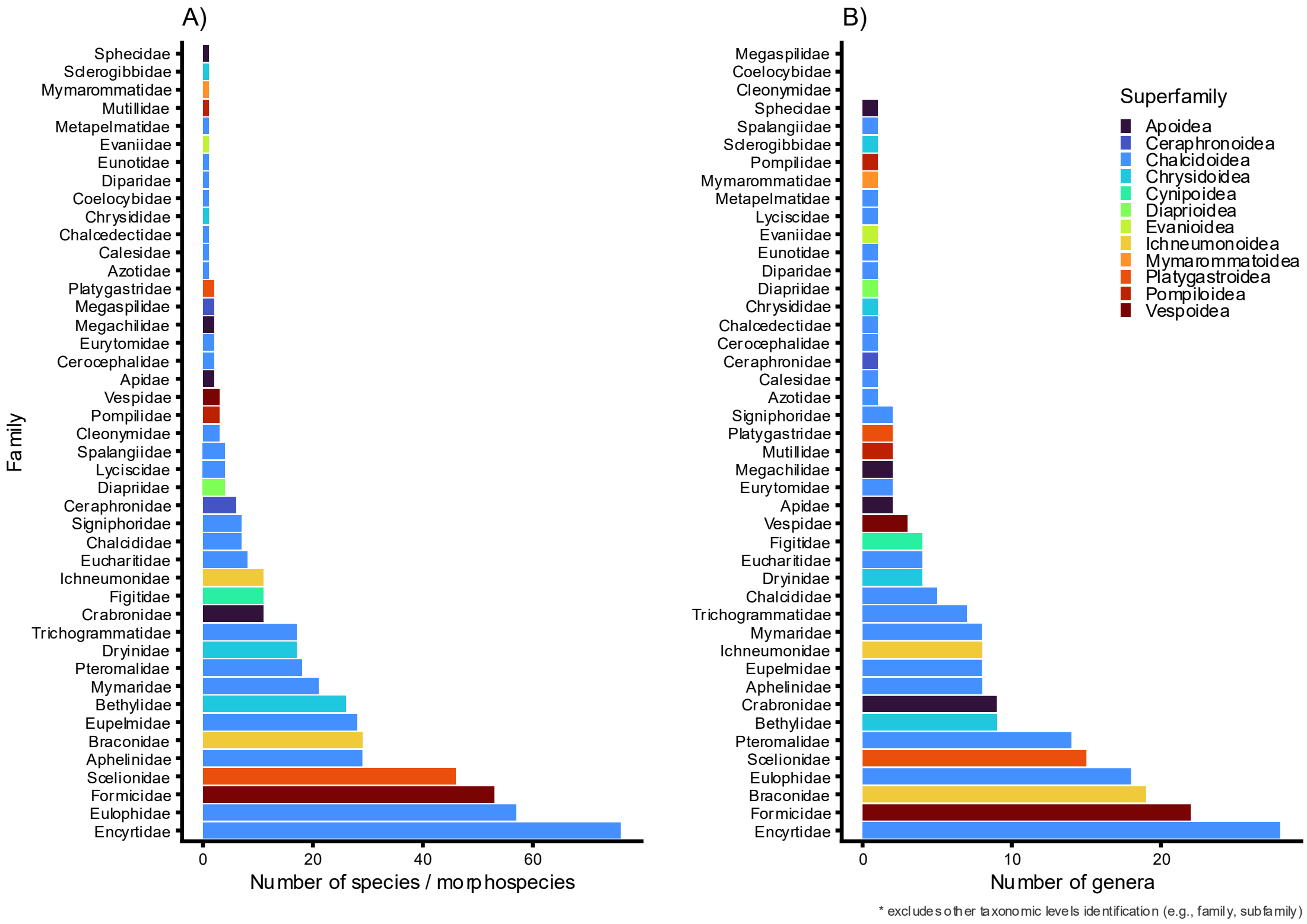

Figure S4. A. Number of species/subspecies/morphospecies per hymenopteran family. B. Number of genera per Hymenoptera family, categorized by superfamily. Each color represents a superfamily. Panel B excludes records identified only at other taxonomic levels, such as family or subfamily.

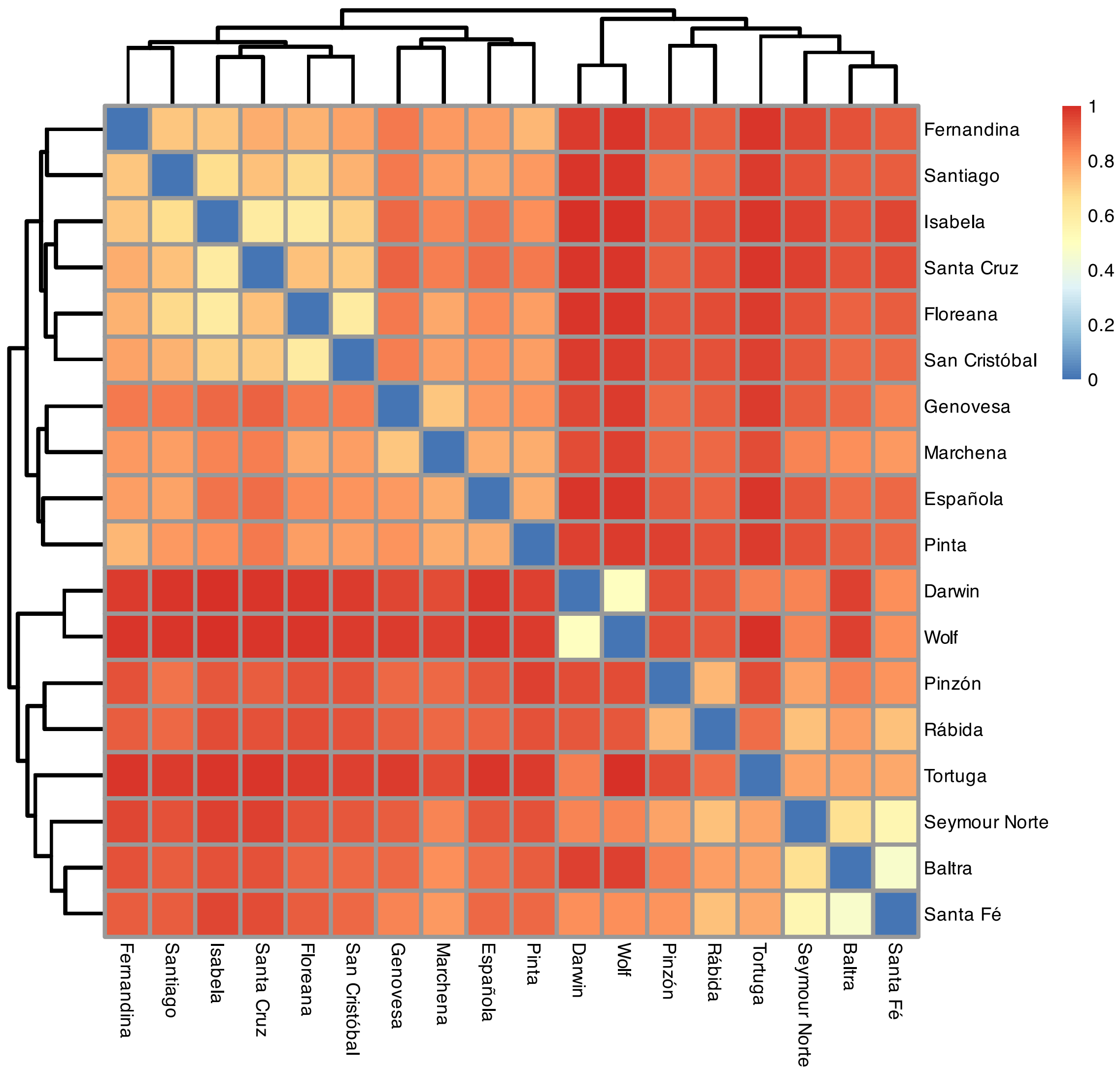

Figure S5. Heat map of the Jaccard dissimilarity distances calculated using the species/morphospecies composition of Hymenoptera from each island of the Galápagos archipelago. Upper and lower diagonals are equivalent. Dendrograms were calculated using complete linkage. Colors indicate the Jaccard dissimilarity values for each pair of islands, with blue indicating lower dissimilarity and red indicating higher dissimilarity.

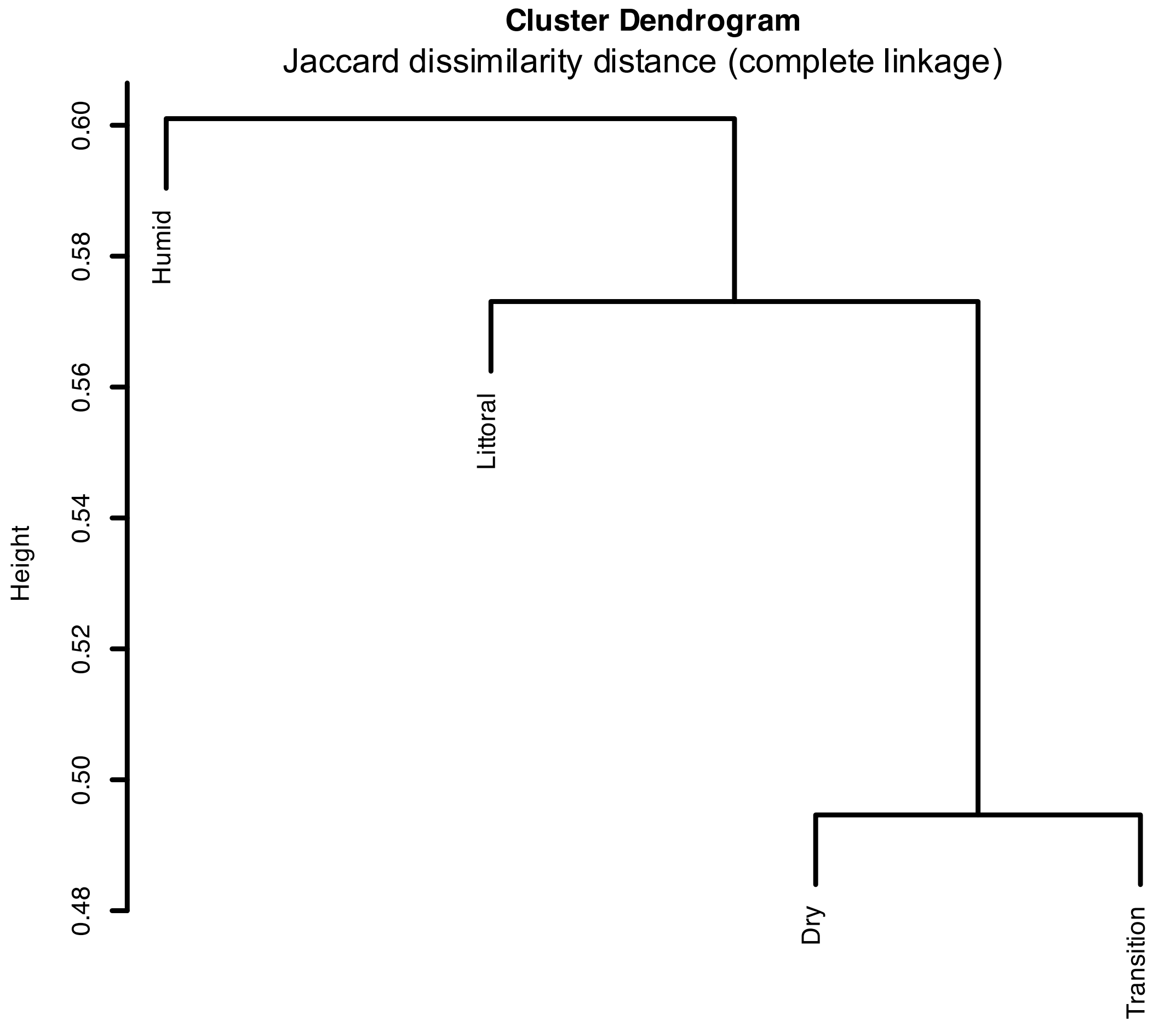

Figure S6. Jaccard dissimilarity distances among vegetation zones, based on species/morphospecies composition. The dendrogram represents hierarchical clustering of the zones using complete linkage.

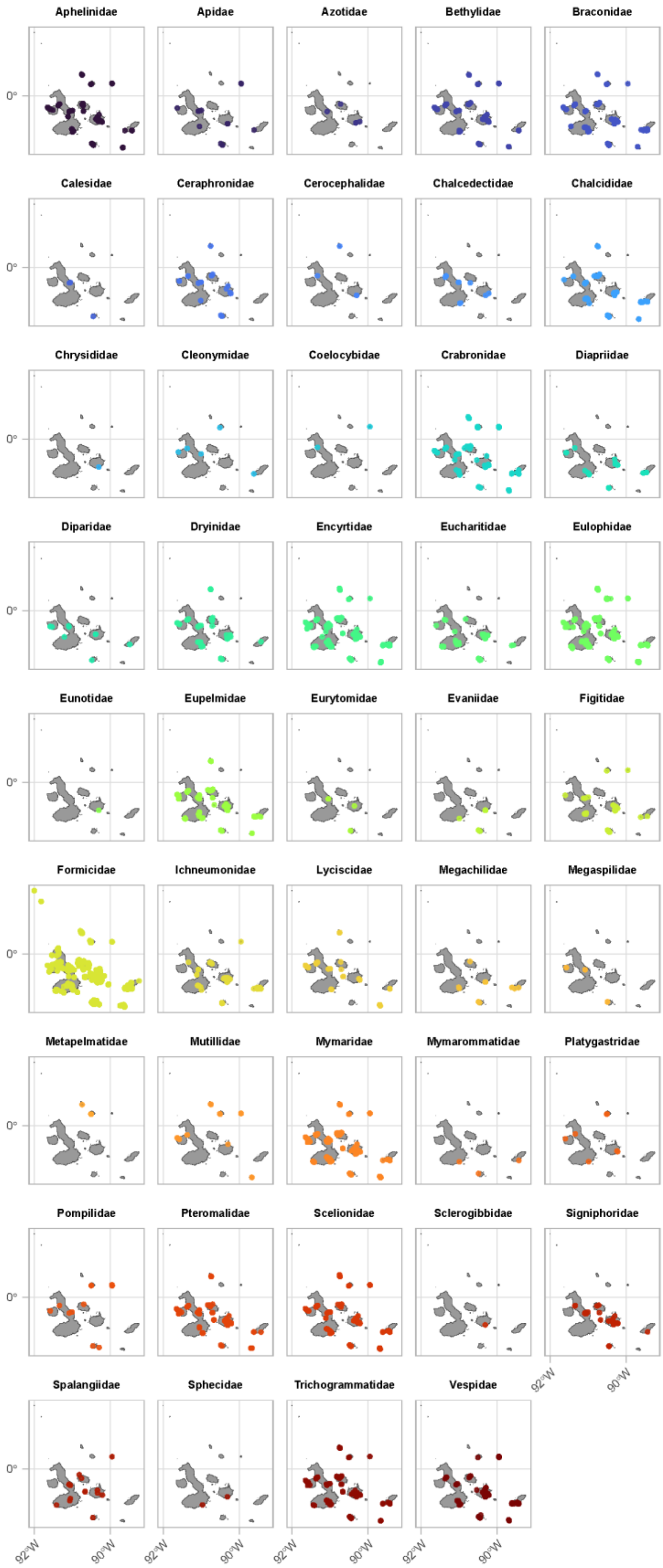

Figure S7. Geographic distribution of hymenopteran families in the Galápagos archipelago across all datasets, based on localities represented by records with geographic coordinates. Country boundaries were obtained from Natural Earth (Admin 0 – Countries, naturalearthdata.com). Maps projected in WGS 84 / UTM Zone 15S.
